# Efficacy and safety of Nefecon, glucocorticoids, or supportive therapyadded to RASi+SGLT2i in IgA nephropathy

**DOI:** 10.3389/fphar.2026.1897246

**Published:** 2026-08-26

**Authors:** Tiantian Zhou, Hao Zhang, Shangchao Liu, Yongqiang Ji, Wei Lv

**Affiliations:** 1 School of Clinical Medicine, Shandong Second Medical University, Weifang, Shandong, China; 2 Yantaishan Hospital, Yantai, Shandong, China; 3 Department of Nephrology, Yantai Yuhuangding Hospital Affiliated to Qingdao University, Yantai, China; 4 Physical Examination Center, Yantai Yuhuangding Hospital Affiliated to Qingdao University, Yantai, China

**Keywords:** glucocorticoid, IgAN, Nefecon, SGLT 2 inhibitor, urinary protein

## Abstract

**Background:**

IgAN is the most common primary glomerulonephritis worldwide. Currently, “RASi + SGLT2i” has become the standard basic treatment regimen. Nefecon is an oral budesonide formulation targeting the intestinal mucosa.

**Methods:**

A single-center retrospective cohort study was conducted, including patients with eGFR >30 mL/min/1.73 m^2^ and proteinuria >0.5 g/d. The basic treatment group received RASi + SGLT2i, the glucocorticoids group received additional glucocorticoids, and the Nefecon group received 16 mg/d of Nefecon. The treatment lasted for 9 months. The primary endpoint was the change in 24-h urine protein.

**Results:**

The 24-h UPro in all three groups decreased significantly from baseline, and there was no statistically significant difference in the reduction among the groups (P = 0.897). The reduction in the Nefecon group (−59.9%) was similar to that of the glucocorticoids group (−46.5%), and it took effect faster (3 months vs. 6 months). The baseline eGFR of the Nefecon group was lower but remained stable during treatment. Clinical remission rate (urine protein reduction ≥50%): the basic treatment group 41.8%, the glucocorticoids group 52.4%, and the Nefecon group 70.0% (P = 0.018), but the baseline urine protein of the Nefecon group was higher, and the results were biased. There was no difference in the incidence of adverse reactions between the Nefecon group and the glucocorticoids group (50.0% vs. 45.2%, P = 0.52), and the infection rates were 5.0% and 9.5% respectively (P > 0.05). However, isolated leukocyte elevation was more common in the Nefecon group (25.0% vs. 9.5%).

**Conclusion:**

The effect of RASi + SGLT2i combined with Nefecon in reducing urine protein is similar to that of glucocorticoids, with faster onset and stable renal function. The conclusion is robust after multi-factor correction, but the retrospective design leads to baseline imbalance and limited correction efficacy, which requires verification through a prospective study. Nefecon may be a potential alternative to glucocorticoids, but attention should be paid to the risk of infection during use.

## Introduction

Immunoglobulin A nephropathy (IgAN) is the most common glomerulonephritis (GN) worldwide, with a minimum overall incidence rate of 2.5 per 100,000 population (Wyatt and Julian, 2013). The disease course is highly heterogeneous, with approximately 30%–40% of patients progressing to kidney failure within 10–20 years (Pattrapornpisut et al., 2021; Xie and Chen, 2008). Specific therapies for IgAN are lacking, and some conventional treatments fail to achieve complete disease control. Therefore, seeking more effective disease-modifying therapies is of critical importance.

The pathogenesis of IgAN is closely related to abnormalities in intestinal mucosal immunity—B cells in gut-associated lymphoid tissue produce galactose-deficient IgA1 (Gd-IgA1), which deposits in the kidney, triggering abnormal immune responses that lead to glomerular inflammation and injury. Nefecon (oral budesonide enteric-coated capsule) is the first targeted therapy designed based on the “gut–kidney axis” theory (Barratt et al., 2020). As a locally acting glucocorticoid, it inhibits the abnormal activation of the ileal mucosa and reduces the production of Gd-IgA1 (Barratt et al., 2024), thereby blocking key pathogenic steps in IgAN. The phase II clinical trial (NEFIGAN) (Fellström et al., 2017) showed that after 9 months of treatment, the urine protein-to-creatinine ratio in the 16 mg/d group was significantly reduced by 27.3% from baseline (compared with a 2.7% increase in the placebo group), and the reduction in proteinuria was maintained at 33% after 3 months off treatment. eGFR remained stable in the treatment group but decreased by 9.8% in the placebo group (p = 0.001). The phase III trial (NefIgArd) (Lafayette et al., 2023; Zhang et al., 2024) further confirmed that in high-risk IgAN patients receiving optimized supportive care, Nefecon treatment for 9 months significantly reduced proteinuria and provided sustained long-term kidney function protection even after treatment discontinuation.

RAS inhibitors (RASi) are the cornerstone of background therapy, with evidence demonstrating effective reduction of proteinuria and stabilization of kidney function (Praga et al., 2003). The KDIGO guidelines recommend that for patients with proteinuria >0.5 g/day, RASi should be used even if blood pressure is normal, and titrated to the maximally tolerated dose to achieve proteinuria targets (Floege et al., 2025a). SGLT2 inhibitors (SGLT2i) have provided a major breakthrough in recent years for supportive therapy in IgAN. The DIAMOND study showed that dapagliflozin significantly reduced proteinuria after 6 weeks of treatment, with sustained efficacy after drug discontinuation (Cherney et al., 2020). Furthermore, the DAPA-CKD trial demonstrated that adding dapagliflozin reduced the risk of kidney endpoints in IgAN, slowed the decline in kidney function, and improved patients’ quality of life (Wheeler et al., 2021; Heerspink et al., 2021). Consequently, KDIGO recommends the dual combination of “RASi + SGLT2i” as background therapy for IgAN (Floege et al., 2025a).

Glucocorticoids are the most widely used immunosuppressive agents. The TESTING study, as a pivotal randomized controlled trial, confirmed that adding methylprednisolone to optimized supportive care significantly reduces proteinuria, slows eGFR decline, and significantly lowers serum Gd-IgA1 levels (Lv et al., 2017). However, to date, there are no real-world comparative reports or data on “RASi + SGLT2i + Nefecon” versus “RASi + SGLT2i + steroids” for the treatment of IgAN. Based on the above information, Nefecon may improve outcomes in IgAN patients. Therefore, we designed and conducted an observational cohort study to evaluate and compare the efficacy and safety of adding Nefecon, steroids, or supportive therapy alone to the “RASi + SGLT2i” backbone for the treatment of IgA nephropathy.

## Materials and methods

### Patient selection

This study was a single-center retrospective cohort study. We collected patients who visited the Nephrology Department of Yantai Yuhuangding Hospital from August 2024 to June 2025 and met the inclusion criteria: primary IgA nephropathy confirmed by renal biopsy, age over 18 years old, estimated eGFR >30 mL/min/1.73 m^2^ according to the formula of the Chronic Kidney Disease Epidemiology Collaboration in 2009, and despite receiving supportive treatment, 24-h urine protein total still >0.5 g/24 h or having a risk of progressive renal function loss; exclusion criteria included: secondary IgA nephropathy or non-IgA nephritis glomerulonephritis, patients who had undergone kidney transplantation and were receiving dialysis treatment, patients with poorly controlled type 1 or 2 diabetes (glycated hemoglobin [HbA] > 8%), patients with poorly controlled blood pressure (blood pressure remained ≥140/90 mmHg after using antihypertensive drugs), patients with malignant tumors, severe liver diseases, autoimmune diseases, etc., which may affect the research results.

## Research design

### Grouping principle

This study is a non-randomized, retrospective observational study. The treatment regimen was not assigned by the study but determined based on clinical practice records reflecting shared decision-making between patients and physicians. Patients were divided into three groups according to actual medication use documented in retrospective medical records: standard baseline therapy group: “RASi + SGLT2i”; Glucocorticoids group: “RASi + SGLT2i + steroids”; and Nefecon group: “RASi + SGLT2i + Nefecon”. In all cases, RASi + SGLT2i were administered at the maximum tolerated dose acceptable to each patient; Glucocorticoids were given using a tapering regimen—0.4 mg/kg/day (maximum 32 mg/day) for 2 months, followed by a reduction of 4 mg/day per month thereafter; Nefecon was administered at 16 mg once daily for 9 months. Supportive treatments such as antihypertensive, antidiabetic, and diuretic therapies were permitted during treatment, but no other immunosuppressive agents (e.g., mycophenolate mofetil, cyclophosphamide) were allowed.

### Data collection

This study collected the clinical data of the patients, including baseline data (based on the last laboratory test before simple supportive treatment, supportive treatment combined with glucocorticoidss, and supportive treatment combined with Nefecon treatment) and general information and laboratory test results at 3, 6, and 9 months after the start of treatment. The collected general information included gender, age, medical history, smoking and drinking history, and treatment plan. Laboratory tests included serum albumin (ALB), blood glucose, eGFR, creatinine, 24-h urine protein quantification, 24-h urine protein total amount, urine red blood cell (RBC) count, red blood cells, hemoglobin (Hgb), white blood cells, platelet count, and absolute neutrophil count.

### Observation indicators

The primary efficacy endpoint was the change in 24-h urine protein quantification (24 h-UPro), the percentage change from baseline, and the absolute change at 9 months of treatment. The secondary efficacy endpoints were the changes in eGFR, creatinine, and urine red blood cells from baseline at 9 months of treatment; and the clinical remission rate at 9 months of treatment (defined as the proportion of patients with a ≥50% reduction in urine protein).

### Efficacy assessment

Adverse events (AEs) and serious adverse events (SAEs) were assessed. The evaluation included infections at any site, respiratory tract infections, urinary tract infections, hypotension, Cushing’s syndrome, arthralgia, abnormal glucose tolerance, and weight gain. Serious adverse events (SAEs) were defined as death from any cause and other life-threatening conditions requiring hospitalization.

### Statistical methods

For continuous variables, normally distributed data are presented as mean ± standard deviation (SD), and non-normally distributed data as median [interquartile range (IQR)]. Comparisons between two groups were performed using the independent samples t-test (for normally distributed data) or the Wilcoxon rank-sum test (for non-normally distributed data). Comparisons among multiple groups were performed using one-way ANOVA (for normally distributed data with homogeneity of variance) or the Kruskal–Wallis H test (for non-normally distributed data), with *post hoc* pairwise comparisons conducted using LSD or Bonferroni correction. Differences in clinical remission rates at 9 months among the three groups were compared using the chi-square test, and intergroup comparisons were performed using the log-rank test; Fisher’s exact test was used when expected counts were insufficient. A P value < 0.05 was considered statistically significant.

## Results

### Baseline characteristics

This study included a total of 165 patients with IgA nephropathy (IgAN). They were divided into three groups according to the treatment plan: the supportive group (73 patients), the glucocorticoids group (47 patients), and theNefecon group (45 patients). During the study, some patients dropped out due to loss to follow-up, data loss, etc. Eventually, 149 patients (67 in the supportive group, 42 in the glucocorticoids group, and 40 in theNefecon group) completed a complete 9-month follow-up and were included in the statistics. The supportive group only received basic supportive treatment (RASi + SGLT2i, treatment with the maximum allowable dose of RAS inhibitors); the glucocorticoids group received additional glucocorticoids treatment on top of the supportive treatment; theNefecon group received additionalNefecon treatment on top of the supportive treatment. All patients completed at least 9 months of treatment. The baseline characteristics of all patients are shown in Table 1.

**TABLE 1:** Main characteristics at baseline.

Indicators	Support group (n = 67)	Glucocorticoids group (n = 42)	Nefecon group (n = 40)	P Value
Gender	​	​	​	0.798
Male [n (%)]	31 (46.3)	21 (50.0)	19 (47.5)	​
Female [n (%)]	36 (53.7)	21 (50.0)	21 (52.5)	​
Age (years)	46.92 ± 12.48	44.52 ± 11.83	46.55 ± 11.56	0.748
Hypertension [n (%)]	​	​	​	0.313
Yes	29 (43.3)	15 (35.7)	18 (45.0)	​
No	38 (56.7)	27 (64.3)	22 (55.0)	​
Diabetes mellitus [n (%)]	​	​	​	0.942
Yes	7 (10.4)	5 (11.9)	4 (10.0)	​
No	60 (89.6)	37 (88.1)	36 (90.0)	​
Smoking [n (%)]	​	​	​	0.146
Yes	4 (6.0)	3 (7.1)	6 (15.0)	​
No	63 (94.0)	39 (92.9)	34 (85.0)	​
Alcohol consumption [n (%)]	​	​	​	0.268
Yes	3 (4.5)	1 (2.4)	4 (10.0)	​
No	64 (95.5)	41 (97.6)	36 (90.0)	​
Hemoglobin (g/L)	137.95 ± 19.78	140.52 ± 18.76	136.89 ± 21.45	0.677
Platelets (×109/L)	248.62 ± 63.15	252.17 ± 60.89	245.98 ± 66.72	0.853
White blood cells (×109/L)	7.33 ± 2.67	7.56 ± 2.74	7.45 ± 2.78	0.923
Albumin (g/L)	40.15 ± 4.61	40.58 ± 4.37	40.02 ± 4.75	0.802
Fasting blood glucose (mmol/L)	5.19 ± 0.94	5.17 ± 0.90	5.15 ± 0.88	0.934
Serum creatinine (μmol/L)	103.18 ± 45.52	90.64 ± 41.13	105.87 ± 51.32	0.150
Estimated glomerular filtration rate (ml/min/1.73m2)	76.58 ± 29.08	86.64 ± 24.35	75.23 ± 28.76	0.166
24-h urinary protein quantification (g/d)	0.84 ± 0.89	0.82 ± 0.75	0.92 ± 1.15	0.692
24-h proteinuria (g/d), median (IQR)	1.16(IQR 0.56–1.19)	1.66(IQR 0.60–1.73)	2.42(IQR 1.29–2.78)	0.689
Urinary red blood cell count (/μl)	104.81 ± 205.68	102.64 ± 187.47	98.56 ± 168.34	0.877
Oxford classification				
M 0/1	44/23	23/19	22/18	0.408
E 0/1	37/30	20/22	22/18	0.704
S 0/1	30/37	22/20	20/20	0.726
T 0/1/2	50/17/0	27/14/1	26/11/3	0.416
C 0/1/2	39/27/1	21/17/3	27/12/1	0.347

### Primary outcomes

**FIGURE 1:**
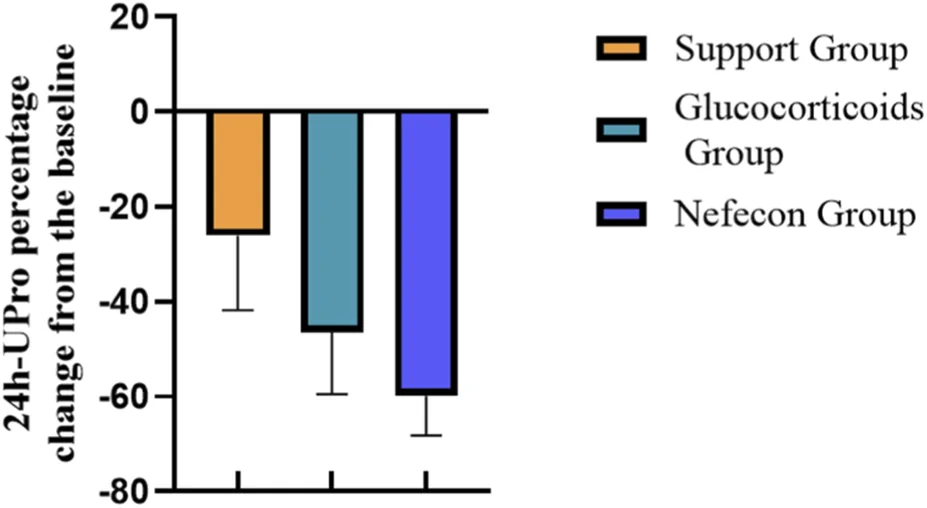
At 9 months of treatment, the percentage change in 24-h urine protein quantification (24 h-UPro) compared to the baseline. The bars represent the median of the percentage decrease in each group, and the error bars represent the 95% confidence interval (CI). The 24 h-UPro of the three groups of patients at 9 months was significantly lower than the baseline (all P < 0.01).

**FIGURE 2:**
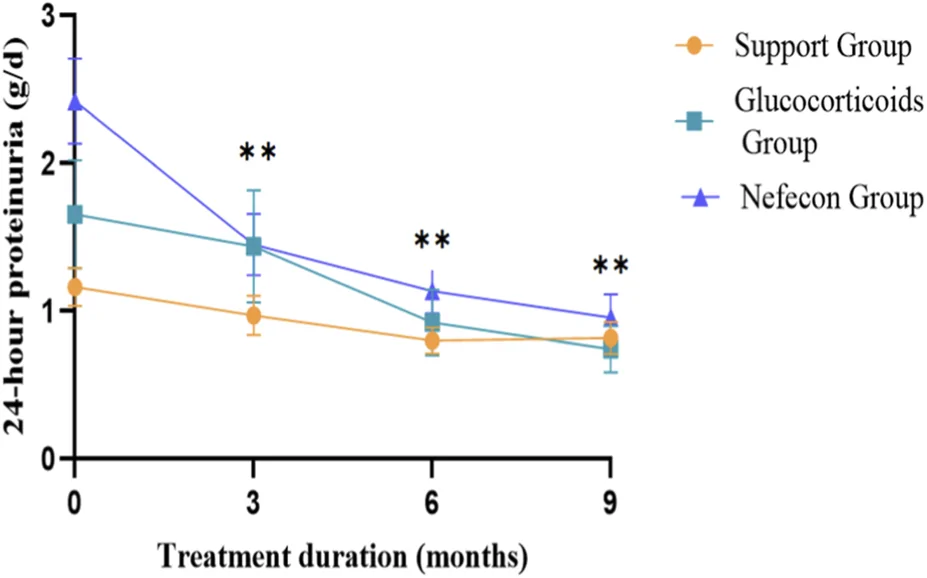
The 24-h urine protein quantification (24 h-UPro) levels during the follow-up period were measured in the Support group, the Glucocorticoids group, and the Nefecon group. The points represent the median of 24 h-UPro, and the error bars indicate the 25th and 75th percentiles (IQR). Within the Nefecon group, comparisons at each time point with the baseline were made, with *P < 0.05 and **P < 0.01; there was no statistically significant difference in 24 h-UPro levels among the three groups (P > 0.05).

At 9 months of treatment, in the standard background therapy group, 24 h-UPro decreased from 1.16 (IQR 0.56–1.19) g to 0.82 (IQR 0.30–0.86) g, representing a mean reduction of 26.0% from baseline (P < 0.001). In the steroid group, 24 h-UPro decreased from 1.66 (IQR 0.60–1.73) g to 0.74 (IQR 0.21–0.73) g, a mean reduction of 46.5% (P = 0.001). In the Nefecon group, 24 h-UPro decreased from 2.42 (IQR 1.29–2.78) g to 0.96 (IQR 0.26–1.27) g, a mean reduction of 59.9% (P < 0.001) (Figures 1, 2). The difference in the magnitude of reduction in 24 h-UPro from baseline among the three groups was not statistically significant (F = 0.109, P = 0.897). Pairwise comparisons showed that the reduction in the Nefecon group (−59.9%) was significantly greater than that in the standard background therapy group (−26.0%, P = 0.001), but the difference compared with the steroid group (−46.5%) was not statistically significant (P = 0.233).

**FIGURE 3:**
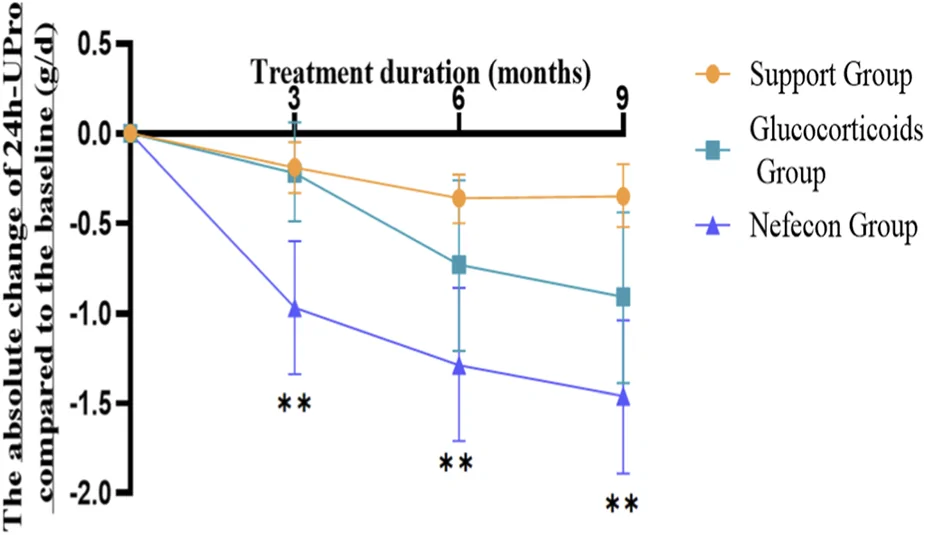
The mean absolute change in 24-h urine protein quantification (24 h-UPro) compared to the baseline for the three groups over the follow-up period is shown in the graph. The points represent the mean absolute change from the baseline, and the error bars represent the 95% confidence interval (CI). Within the Nefecon group, comparisons at each time point with the baseline, *P < 0.05, **P < 0.01, compared with the baseline of this group.

In terms of absolute changes, at 3, 6, and 9 months of treatment, the reductions in 24 h-UPro from baseline were 0.19 g/d, 0.36 g/d, and 0.35 g/d, respectively, in the standard background therapy group; 0.22 g/d, 0.73 g/d, and 0.91 g/d, respectively, in the steroid group; and 0.97 g/d, 1.29 g/d, and 1.46 g/d, respectively, in the Nefecon group. Among them, the Nefecon group had the fastest onset of action, achieving a significant reduction as early as 3 months of treatment, and maintained a greater reduction throughout the follow-up period compared with both the steroid group and the standard background therapy group. In contrast, the steroid group showed only a modest reduction at 3 months (not statistically significant) and did not achieve a significant reduction until 6 months (Figure 3).

### Secondary outcomes

**FIGURE 4:**
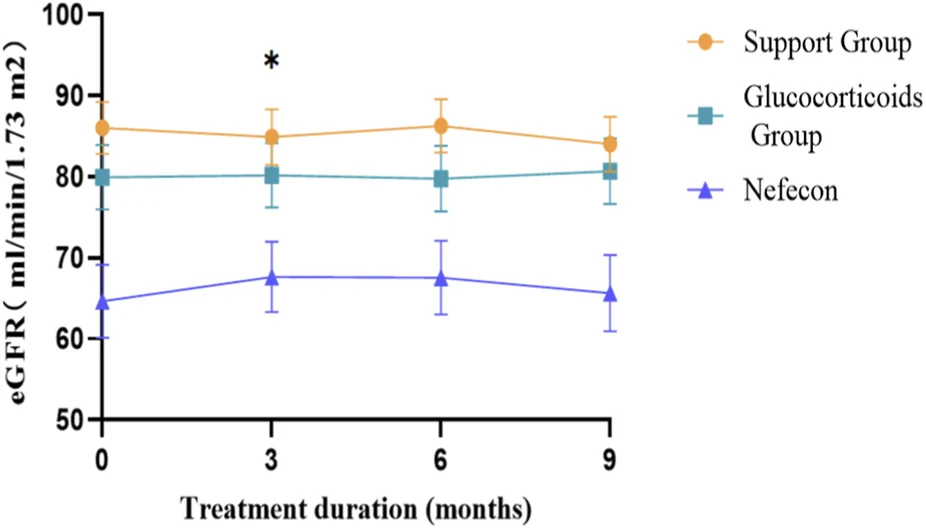
The changes in glomerular filtration rate (eGFR) levels during the follow-up period were observed in the Support group, the Glucocorticoids group, and the Nefecon group. The dots represent the mean absolute changes from the baseline, and the error bars represent the 95% confidence interval (CI). Within the Nefecon group, comparisons at each time point with the baseline, *P < 0.05, **P < 0.01, were made against the baseline of this group.

The support group decreased from 86.00 ± 26.42 mL/min/1.73 m^2^ to 84.02 ± 27.60 mL/min/1.73 m^2^ (P = 0.042); the glucocorticoids group decreased from 80.15 ± 26.34 mL/min/1.73 m^2^ to 82.20 ± 24.99 mL/min/1.73 m^2^ (P = 0.234); the Nefecon group decreased from 64.67 ± 28.47 mL/min/1.73 m^2^ to 65.65 ± 29.65 mL/min/1.73 m^2^ (P = 0.473). There was no statistically significant difference in eGFR among the three groups at baseline and 9 months (all P > 0.05), and the trend of renal function change was consistent, with no significant progressive decline (Figure 4).

Baseline eGFR was significantly lower in the Nefecon group than in the steroid group (64.67 vs. 80.15 mL/min/1.73 m^2^, P = 0.012). However, during the 9-month treatment period, eGFR remained stable in the Nefecon group (P = 0.473), with no further deterioration.

**FIGURE 5:**
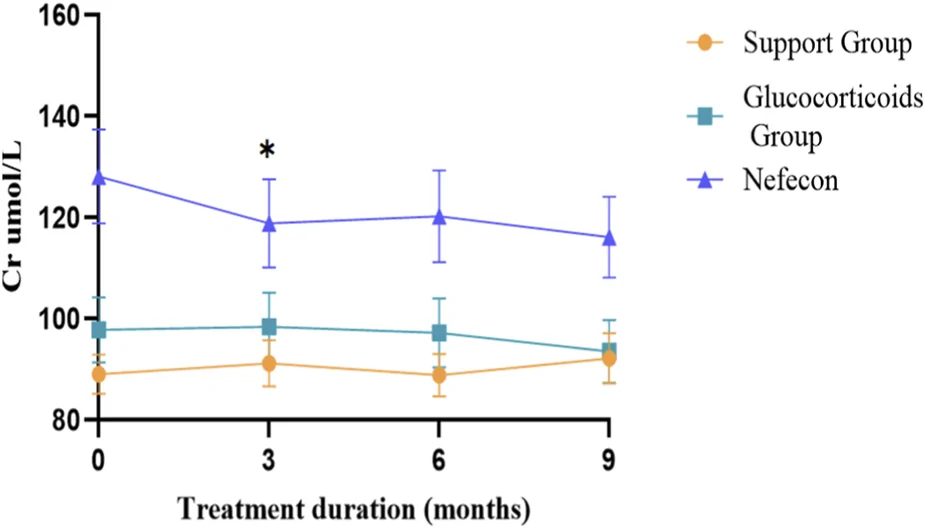
The changes in creatinine Cr levels during the follow-up period were observed in the Support group, the Glucocorticoids group, and the Nefecon group. The points represent the mean absolute change from the baseline, and the error bars represent the 95% confidence interval (CI). Within the Nefecon group, comparisons at each time point with the baseline, *P < 0.05, **P < 0.01, were made against the baseline of this group.

The 9-month Cr level in the Nefecon group decreased from 128.15 ± 58.89 μmol/L to 127.70 ± 73.25 μmol/L (P = 0.931), and then transiently dropped to 118.93 ± 55.37 μmol/L at 3 months (P = 0.001), and recovered to 122.83 ± 62.92 μmol/L at 6 months (P = 0.161), showing overall stability. The Cr level in the Support group changed from 88.45 ± 31.72 μmol/L to 91.75 ± 40.87 μmol/L (P = 0.103), while that in the glucocorticoids group decreased from 97.83 ± 41.47 μmol/L to 93.55 ± 40.34 μmol/L (P = 0.114). There was no statistically significant difference in Cr levels between the Support group and the glucocorticoids group at each time point (all P > 0.05); while there were statistically significant differences between the Nefecon group and the Support group, and between the Nefecon group and the glucocorticoids group at baseline, 3 months, 6 months, and 9 months (all P < 0.05). This difference was caused by the different baseline renal function levels. During the follow-up period, the Cr change trends of the three groups were consistent, suggesting good renal safety for all (Figure 5).

Comparisons of serum creatinine levels between the glucocorticoids group and the Nefecon group at baseline (P = 0.008), 3 months (P = 0.066), 6 months (P = 0.036), and 9 months (P = 0.010) showed statistically significant differences at baseline, 6 months, and 9 months, but not at 3 months.

**FIGURE 6:**
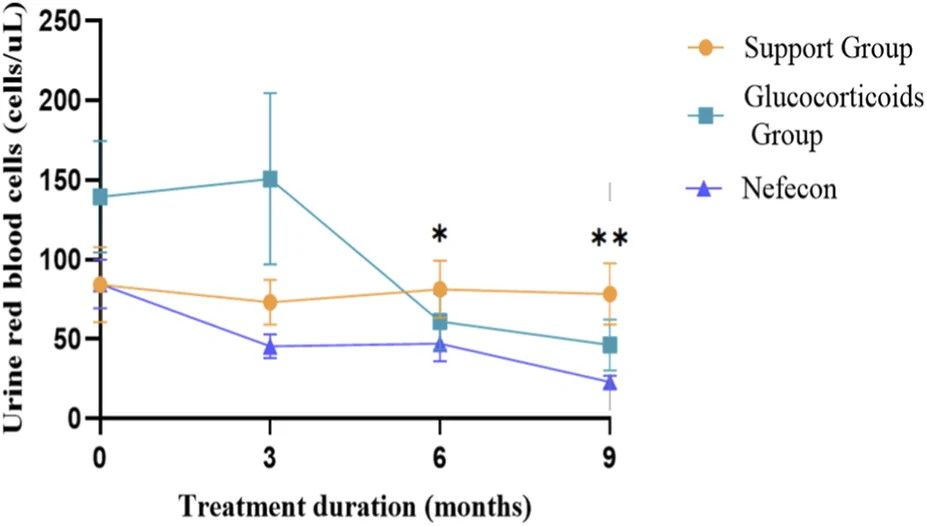
The changes in urine red blood cell levels during the follow-up period were observed in the Support group, the Glucocorticoids group, and the Nefecon group. The dots represent the mean absolute change from the baseline, and the error bars represent the 95% confidence interval (CI). Within the Nefecon group, comparisons at each time point with the baseline, *P < 0.05, **P < 0.01, were made against the baseline of this group.

The 9-month urine RBC count in the Nefecon group decreased from 84.74 cells/μL (IQR: 8.08–68.33) to 22.97 cells/μL (IQR: 6.48–43.28) (P = 0.000); the Support group decreased from 83.67 cells/μL (IQR: 8.08–68.33) to 77.31 cells/μL (IQR: 6.48–43.28) (P = 0.819); the glucocorticoids group decreased from 134.71 cells/μL (IQR: 8.08–68.33) to 47.13 cells/μL (IQR: 6.48–43.28) (P = 0.018). At 9 months of treatment, the urine RBC counts of all three groups showed a downward trend, with the most significant decrease in the glucocorticoids group, followed by the Nefecon group, and no significant fluctuation in the Support group. The only statistically significant difference was between the Nefecon group and the Support group at 9 months (P = 0.025); the difference between the glucocorticoids group and the Nefecon group was statistically significant at 3 months (P = 0.047), and no statistical significance was observed at other time points (all P > 0.05) (Figure 6).

Comparisons of urine RBC count levels between the glucocorticoids group and the Nefecon group at baseline (P = 0.221), 3 months (P = 0.047), 6 months (P = 0.371), and 9 months (P = 0.367) showed statistically significant differences only at 3 months, and no statistical significance at other time points.

### Treatment efficacy

There was no statistically significant difference in the clinical remission rate of ≥50% reduction in urine protein between the three groups at 3 months (P = 0.206) and 6 months (P = 0.098) of treatment. However, at 9 months of treatment, the difference was statistically significant (P = 0.018). At 9 months, the clinical remission rates of ≥50% reduction in urine protein for the three groups were as follows: 41.8% (28/67) in the support group, 52.4% (22/42) in the glucocorticoids group, and 70.0% (28/40) in the Nefecon group (Table 2). Further analysis using Kaplan-Meier survival analysis compared the cumulative remission rates among the three groups, and the difference was statistically significant (log-rank test, χ^2^ = 7.049, P = 0.029).

**TABLE 2:** Comparison of the clinical remission rates (where urine protein decreased by ≥ 50% at 3, 6, and 9 months of treatment) among the three groups of patients.

Group	Time	Time point remission rate (cross-sectional)	KM cumulative remission rate
Support group	3 months	22.4% (15/67)	22.4%
	6 months	32.8% (22/67)	40.3%
	9 months	41.8% (28/67)	50.7%
Glucocorticoids group	3 months	33.3% (14/42)	33.3%
	6 months	47.6% (20/42)	52.4%
	9 months	52.4% (22/42)	64.3%
Nefecon group	3 months	37.5% (15/40)	37.5%
	6 months	52.5% (21/40)	55.0%
	9 months	70.0% (28/40)	77.5%

There was a statistically significant difference in the inter-group comparison of urine protein reduction (F = 6.002, P = 0.003). Among them, the reduction amplitude of the Nefecon group (−59.9%) was significantly greater than that of the support group (−26.0%, P = 0.001), but there was no statistically significant difference compared with the glucocorticoids group (−46.5%, P = 0.233). At the same time, the proportion of patients achieving ≥50% reduction in the Nefecon group was also significantly higher than that of the glucocorticoids group and the support group (70.0% vs. 52.4% vs. 41.8%, P = 0.018). This may be partly due to the highest baseline proteinuria level in the Nefecon group (2.42 vs. 1.66 g/d), which makes it easier to reach the 50% relative threshold with the same absolute reduction; from the absolute reduction perspective, the absolute value of urine protein in the Nefecon group decreased by 1.46 g/d at 9 months, which was also higher than that of the glucocorticoids group (0.91 g/d) and the support group (0.34 g/d), suggesting that Nefecon has a stronger protein-lowering effect. At the same time, it is also not excluded that the individual therapeutic effect of Nefecon is more consistent. This difference is one of the important reasons why the clinical remission rate of the Nefecon group was significantly higher than that of the other two groups.

### Safety and AEs

During the follow-up period, all groups had good tolerance. The total incidence of adverse reactions in the Nefecon group and the glucocorticoids group was 50.0% (20/40) and 45.2% (19/42) respectively, both higher than that of the supportive control group (6.7%, 2/30), and the difference was statistically significant (P < 0.001). There was no statistically significant difference between the two groups (P > 0.05). The adverse events were mainly grade 1–2 according to CTCAE. There were no events of ≥3 grades or serious adverse events, and no patients stopped the treatment due to adverse events. In the Nefecon group, there were 2 cases of infection (5.0%), 10 cases of isolated leukocytosis (25.0%); in the glucocorticoids group, there were 4 cases of infection (9.5%), 4 cases of isolated leukocytosis (9.5%). The infection rate of the Nefecon group was numerically lower than that of the hormone group, but isolated leukocytosis was more common. It is recommended to strengthen the monitoring of blood routine (Table 3).

**TABLE 3:** The incidence of adverse reactions in the three groups of patients, with a p-value indicating a statistically significant difference between the Nefecon group and the Glucocorticoids group.

Adverse reactions, n%	Support group (n = 67)	Glucocorticoids group (n = 42)	Nefecon group (n = 40)	P value
Infection	2 (3.0)	4 (9.5)	2 (5.0)	​
Simple increase in white blood cells	​	3 (7.1)	10 (25.0)	​
High blood sugar	1 (1.5)	3 (7.1)	4 (10.0)	​
Anemia	1 (1.5)	4 (9.5)	3 (7.5)	​
Menstrual disorders	​	​	1 (2.5)	​
Obesity	​	2 (4.8)	​	​
Edema	​	1 (2.4)	​	​
Dizziness	​	1 (2.4)	​	​
Incidence of AEs	4 (6.7%)	18 (42.9%)	20 (50%)	0.52

### Sensitivity analysis

To adjust for baseline differences between groups (the Nefecon group had higher baseline proteinuria and lower eGFR), we used a multivariable linear regression model. After adjustment, the reductions in urinary protein were −26.01%, −46.54%, and −59.90% across the groups, nearly identical to the unadjusted results (−26.0%, −46.5%, −59.9%). This high consistency suggests that baseline imbalances did not substantially confound the main conclusions, indicating robustness of the findings. The near-identical estimates are explained by the limited explanatory power of the included covariates rather than by insufficient adjustment. Due to the observational study design, these conclusions still require validation through prospective studies (Table 4). The full regression output, including coefficients, 95% CIs, model R^2^, and collinearity diagnostics (VIF), is provided in Supplementary Tables S1, S2.

**TABLE 4:** Comparison of proteinuria reduction among the three groups after adjusting for baseline differences between groups.

Group	Unadjusted reduction (%)	Adjusted reduction (%)
Support group (n = 67)	−26.0%	−26.01%
Glucocorticoids Group (n = 42)	−46.5%	−46.54%
Nefecon group (n = 4)	−59.9%	−59.90%

## Discussion

For many years, IgAN has remained the most common primary glomerular disease worldwide, particularly in East Asian countries. The disease presents with diverse manifestations. In China, IgAN accounts for more than half of all primary glomerular diseases and is one of the leading causes of end-stage kidney disease (ESKD) (Rodrigues et al., 2017). Although some patients have a long-term stable clinical course, a substantial proportion still progress to ESKD within 10 years of diagnosis (Berthoux et al., 2011; Li et al., 2023). Currently, the underlying mechanisms of IgAN have been thoroughly elucidated at the submolecular level, with the key finding being the presence of immune complexes containing specific O-glycoforms of IgA1 (Pattrapornpisut et al., 2021; Chang and Li, 2020). These complexes play a critical role in the pathogenesis of the disease. Advances based on the “four-hit hypothesis” have driven the development of various therapeutic approaches, including multiple pharmacologic agents (El Karoui et al., 2024).

Nefecon was conditionally approved for marketing in China in 2023 and included in the national medical insurance (Class B) in 2025, significantly reducing its monthly cost from approximately 23,000 yuan at launch. Taking Yantai City’s policy as an example, combined with initial out-of-pocket payments and reimbursement for outpatient chronic and special diseases, patients’out-of-pocket expenses have decreased substantially, making treatment strategies incorporating Nefecon more practically feasible. However, further pharmacoeconomic studies are needed to evaluate regional differences in cost-effectiveness across various parts of the country.

Nefecon (Budesonide extended-release capsules) is the world’s first targeted intestinal mucosal immunomodulator approved for treating IgA nephropathy (IgAN) (Lin et al., 2025). Its core mechanism is based on the “gut-kidney axis” theory, utilizing a dual delayed-and sustained-release formulation to precisely deliver the drug to the terminal ileal Peyer’s patches (Grimm et al., 2024), where it locally suppresses mucosal B-cell activation at high concentrations, thereby reducing the production of galactose-deficient IgA1 (Gd-IgA1) (Zhai et al., 2026; Barratt et al., 2023). The phase III NefIgArd trial has confirmed that this mechanism translates into sustained reductions in proteinuria and renal function protection (Lafayette et al., 2023). This source-level intervention strategy bypasses the indirect, systemic route through which conventional corticosteroids must circulate before reaching the kidneys. Pharmacokinetically, Nefecon exhibits an absorption lag time of about 3 h, ensuring accurate delivery to the ileum before release begins. Although it achieves relatively high local peak concentrations (6,260 pg/mL at a 16 mg dose), its terminal half-life is only approximately 4.4 h, and around 80%–90% undergoes rapid first-pass hepatic metabolism and inactivation, resulting in minimal systemic exposure and thus fast local action with limited systemic interference (Barratt et al., 2024). However, recent advances in the “gut microbiota metabolic reprogramming” hypothesis suggest that alterations in microbial community structure and metabolic functions can drive early changes in host immune homeostasis via the “microbiome-metabolism-immunity axis,” occurring even prior to host metabolic abnormalities (Wang et al., 2026). Given that gut microbes and their metabolites—such as short-chain fatty acids, bile acids, and tryptophan derivatives—regulate B-cell differentiation and IgA class switching, Nefecon’s effects may extend beyond localized immunosuppression to include modulation of the intestinal microbiome and reshaping of microbial composition. Additionally, the small amount of budesonide absorbed into the bloodstream may mediate limited systemic immune regulation, creating a dual mode of action characterized by “local targeting as primary and systemic exposure as secondary” (Lafayette et al., 2023). This dual mechanism aligns with the recently proposed “dual-pathway” framework:drugs can exert systemic effects via intestinal absorption while simultaneously modulating the gut microbiota and its metabolism to indirectly influence host immunity, synergistically producing multi-target effects. Currently, direct evidence regarding Nefecon’s specific impact on the intestinal microbiome remains limited, and its effects on microbial composition and function warrant further investigation. Clinical studies have also shown significant reductions in serum Gd-IgA1 and IgA-IgG immune complex levels following Nefecon treatment (Nawaz et al., 2026; Wimbury et al., 2024), further supporting its efficacy in targeting the root cause of pathogenic IgA production.

It is worth noting that SGLT2 inhibitors (SGLT2i) and Nefecon may have potential synergistic mechanisms at the intestinal level. In addition to inhibiting glucose reabsorption in the proximal renal tubules, recent studies have shown that SGLT2i significantly affect the composition and function of the gut microbiota:they can reshape the gut microbial structure, enrich short-chain fatty acid (SCFA) producing bacteria, suppress microorganisms that generate toxins such as TMAO, restore intestinal tight junction integrity, and modulate bile acid metabolism (Tao et al., 2025). Animal experiments further indicate that empagliflozin maintains intestinal homeostasis by regulating gut microbiota diversity and tryptophan metabolism (Popova et al., 2025). IgAN patients already exhibit dysbiosis—reduced abundance of butyrate-producing bacteria (such as Butyrococcus and Agathobacter rectalis), and abnormal activation of nucleotide and nucleoside biosynthesis pathways (Popova et al., 2025). This microbial imbalance may contribute to the production of pathogenic IgA by affecting mucosal B cell differentiation and IgA class switching (Wang et al., 2026). Therefore, it is hypothesized that SGLT2i’s ability to correct gut dysbiosis and restore intestinal barrier function may synergize with Nefecon’s direct inhibition of Gd-IgA1 production by mucosal B cells in the terminal ileum (Barratt et al., 2024; Grimm et al., 2024), creating an additive effect at the shared site of action—the intestine:SGLT2i reshapes the gut microbiome and reduces local intestinal inflammation, thereby creating a more favorable intestinal environment for Nefecon’s targeted immunomodulation; meanwhile, Nefecon directly intervenes at the source of pathogenic IgA production. These two mechanisms complement each other and overlap in their sites of action, suggesting a potential synergistic therapeutic effect (Floege et al., 2025b). However, whether Nefecon directly regulates gut microbiota metabolic pathways, and whether SGLT2i′s modulation of the gut microbiome enhances Nefecon’s efficacy, remains unproven and requires systematic research for validation.

This study compared the efficacy and safety of adding Nefecon, adding steroids, or supportive therapy alone to the background regimen of RASi + SGLT2i for the treatment of IgAN. The results demonstrated that Nefecon achieved clinical efficacy similar to steroids in treating IgAN, significantly reducing 24-h urine protein (24 h-UPro) and maintaining stable eGFR. Patients treated with Nefecon showed a rapid response in proteinuria reduction. Although baseline proteinuria was high (2.42 g/d), 24 h-UPro decreased significantly as early as 3 months of treatment, with an onset of action notably faster than that in the steroid group (which did not achieve a significant reduction until 6 months). Concurrently, the urinary red blood cell count also decreased significantly (P = 0.000), suggesting rapid control of glomerular inflammation. Regarding safety, the adverse event rate was 50.0% in the Nefecon group and 42.9% in the steroid group, with no statistically significant difference between the two groups (P > 0.05). The difference in the magnitude of 24 h-UPro reduction between the two groups was also not statistically significant (P = 0.233). In the primary endpoint analysis at 9 months, the overall difference in the magnitude of 24 h-UPro reduction among the three groups was not statistically significant (P = 0.897), which may be related to the relatively small sample size, large data variability. Multivariable regression analysis showed that baseline eGFR was not independently associated with proteinuria reduction (P = 0.875), suggesting that the null finding was unlikely driven primarily by baseline renal function imbalance. In terms of renal function, patients in the Nefecon group maintained stable kidney function. Although they had the lowest baseline eGFR (64.67 mL/min/1.73 m^2^), no significant decline in eGFR was observed after 9 months of treatment (P = 0.473), whereas the standard background therapy group showed a mild decline in eGFR (P = 0.042). Although it is difficult to assess hard endpoints (such as ESKD or a ≥50% decline in eGFR) during a 9-month follow-up, eGFR remained stable in the Nefecon group (especially for patients with the lowest baseline eGFR), and this mid-term signal of renal function protection is worthy of attention. Regarding serum creatinine levels, the Nefecon group showed a transient decrease at 3 months (P = 0.001), returning to baseline levels by 9 months, indicating overall stability. No significant changes in serum creatinine were observed in the steroid or standard background therapy groups. For urinary red blood cells, the Nefecon group showed a significant decrease after 9 months of treatment (P = 0.000), the steroid group also showed a significant decrease (P = 0.018), while the standard background therapy group showed no significant change (P = 0.819). The difference in the 9-month clinical remission rate (proportion of patients with ≥50% reduction in proteinuria) among the three groups was statistically significant (P = 0.029).

When interpreting the results of this study, it is necessary to objectively recognize the potential interference of baseline imbalance and clinical selection bias on the efficacy comparison. In this study, the baseline renal function of the Nefecon group was worse (eGFR 64.67 mL/min/1.73 m^2^), and the urine protein level was higher (2.42 g/d), while those of the glucocorticoids group were 80.15 mL/min/1.73 m^2^ and 1.66 g/d respectively. This imbalance originated from the clinical doctors’ tendency to use the new targeted drugs for high-risk patients with poor traditional treatment or concerns about glucocorticoids, which belongs to the selective bias in the real world. Nevertheless, the Nefecon group still achieved a comparable reduction in urine protein level (−59.9% vs. −46.5%). Multivariable regression analysis showed that baseline eGFR was not independently associated with proteinuria reduction (P = 0.875), and baseline 24 h-UPro showed only a marginal trend (P = 0.078), suggesting that the measured covariates had limited confounding effects on the efficacy comparison. After multivariate correction, the reduction in urine protein level in each group was highly consistent with the uncorrected results, suggesting that the baseline imbalance had limited interference with the main conclusion, and the results were relatively robust. However, observational studies cannot completely rule out the influence of unmeasured confounding factors (such as intestinal flora status, mucosal immune activity, previous medication history and compliance, etc.), and the conclusion still needs to be further verified by a prospective randomized controlled trial.

Safety data showed a numerically lower infection rate with Nefecon (5.0%, 2/40) versus steroids (9.5%, 4/42), though not statistically significant, likely due to small sample size; however, isolated leukocytosis was significantly more frequent in the Nefecon group (25.0%, 10/40) than in steroids (7.1%, 3/42). Although not classified as infection, this finding may indicate subclinical inflammation or mucosal immune modulation and warrants clinical attention, given that Nefecon targets gut mucosal immunity, potentially affecting barrier function and local homeostasis, which might increase susceptibility to pathogens or trigger non specific inflammatory responses. The link between leukocytosis and infection requires further study. Based on these signals, we propose a stratified management approach. For high risk patients with diabetes, urinalysis should be performed before treatment, with monthly monitoring of WBC, CRP, and glucose during the first 6 months; elderly patients (≥65 years) should undergo chest CT prior to Nefecon initiation, have biweekly follow up visits during the first 3 months, and be considered for influenza or pneumococcal vaccination; those with baseline WBC >9.5 × 10^9^/L should delay start by 1–2 weeks and obtain chest CT to rule out occult infection. For post infection management, mild to moderate infections (e.g., upper respiratory tract infections, cystitis) allow continued Nefecon therapy alongside antimicrobials, with reassessment after 3–5 days, whereas severe infections (e.g., pneumonia, pyelonephritis) require temporary discontinuation of Nefecon until infection control is achieved, followed by multidisciplinary evaluation for possible resumption at a reduced dose (8 mg/d). For patients with persistent isolated leukocytosis lasting more than 2 weeks, peripheral blood smear and inflammatory markers (ESR, CRP, IL-6) should be evaluated, and infection risk should be dynamically assessed with adjusted monitoring frequency as needed.

This study has certain limitations. The retrospective, single-center design and limited sample size of this study limited its universality. It may also weaken the statistical power of the multi-factor correction model, causing the estimated values before and after correction in the sensitivity analysis to be very close. Due to the lack of a strict control group design and the short follow-up time (not extended to 12 months), we could not draw a definite conclusion about long-term renal function protection (such as long-term stability of eGFR). In addition, we did not assess changes in serum Gd-IgA1 and other biomarkers, which limited the in-depth understanding of the mechanism of action. Moreover, the sample size of this study was small, and the results need to be further verified by larger-scale multicenter studies.

## Conclusion

On the basis of the standard RASi + SGLT2i treatment, the combination of Nefecon or glucocorticoids can significantly reduce urinary protein in patients with IgAN, and there is no statistically significant difference in the 9-month reduction of urinary protein among the three groups. The onset speed of Nefecon may be faster, and it can stabilize patients with worse baseline renal function. After correcting for baseline covariates, the estimated reduction values were basically consistent, suggesting that the conclusion has a certain degree of robustness. However, due to the unbalanced baseline caused by the retrospective design and the limited sample size, baseline imbalances may still have introduced bias, which prevented us from drawing a definitive conclusion that Nefecon has a better therapeutic effect than glucocorticoids. This study provides important real-world data for the clinical application of Nefecon, but its final positioning still requires prospective, head-to-head randomized controlled trials to clarify.

## Data Availability

The datasets presented in this article are not readily available because the data that support the findings of this study are available from the first author, upon reasonable request. Requests to access the datasets should be directed to TZ, tzhoutiantiasn@163.com.
