# Correction: Efficacy and safety of Nefecon, glucocorticoids, or supportive therapyadded to RASi+SGLT2i in IgA nephropathy

**DOI:** 10.3389/fphar.2026.2006892

**Published:** 2026-10-06

**Authors:** Tiantian Zhou, Hao Zhang, Shangchao Liu, Yongqiang Ji, Wei Lv

**Affiliations:** 1 School of Clinical Medicine, Shandong Second Medical University, Weifang, Shandong, China; 2 Yantaishan Hospital, Yantai, Shandong, China; 3 Department of Nephrology, Yantai Yuhuangding Hospital Affiliated to Qingdao University, Yantai, China; 4 Physical Examination Center, Yantai Yuhuangding Hospital Affiliated to Qingdao University, Yantai, China

**Keywords:** glucocorticoid, IgAN, Nefecon, SGLT 2 inhibitor, urinary protein

Affiliation “School of Clinical Medicine, Shandong Second Medical University, Weifang, Shandong, China “was incorrectly written in the paper as “Clinical Medical College, Shandong Second Medical University, Weifang, Shandong, China”.

Affiliation “Yantaishan Hospital, Yantai, Shandong, China” was incorrectly written in the paper as “School of Clinical Medicine, Shandong Second Medical University, Weifang, Shandong, China”.

The original version of this article has been updated.

