# Supplementary material for: Efficacy and safety of Nefecon, glucocorticoids, or supportive therapyadded to RASi+SGLT2i in IgA nephropathy
Source: Front Pharmacol. 2026 Aug 26;17:1897246. doi: 10.3389/fphar.2026.1897246 (PMC13535521; doi:10.3389/fphar.2026.1897246)
Supplement: Supplementary file 1 [file DataSheet1.pdf]

降幅 Variables
尿蛋白总量 Baseline 24h-UPro
滤过率 Baseline eGFR
年龄 Age
性别 Gender (male vs. female)

Supplementary Table S1. Multivariable linear regression analysis of factors associated with 24h-UPro reduction at 9 months

Variables	Unstandardized Coefficients (B)	Std. Error	Standardized Coefficients (Beta)	t	P value
(Constant)	0.198	0.320	—	0.618	0.538
Baseline 24h-UPro	0.044	0.025	0.152	1.775	0.078
Baseline eGFR	0.000	0.002	0.015	0.158	0.875
Age	0.003	0.004	0.072	0.793	0.429
Gender (male vs. female)	−0.005	0.088	−0.005	−0.061	0.951

Model fit: $R^2 = 0.026$, Adjusted $R^2 = -0.001$, $F = 0.952$, $P = 0.436$.

Dependent variable: Percentage change in 24h-UPro from baseline to 9 months.

Covariates included: Baseline 24h-UPro, baseline eGFR, age, and gender. All variables were entered simultaneously using the Enter method.

Supplementary Table S2. Collinearity diagnostics of covariates in the multivariable linear regression model

Covariate	Tolerance	VIF
Baseline 24h-UPro	0.920	1.087
Baseline eGFR	0.745	1.342
Age	0.830	1.204
Gender	0.940	1.064

Table S2. Variance inflation factor (VIF) values for each covariate. All VIF values were <2, indicating no significant multicollinearity

Regression

[数据集1]

Variables Entered/Removed[a]

Model	Variables Entered	Variables Removed	Method
1	性别，年龄，尿蛋白总量，滤过率[b]	.	Enter

a. Dependent Variable: 降幅

b. All requested variables entered.

Model Summary

Model	R	R Square	Adjusted R Square	Std. Error of the Estimate	Change Statistics	
					R Square Change	F Change
1	0.161[a]	0.026	-0.001	0.52219	0.026	0.952

Model Summary

Model	Change Statistics		
	df1	df2	Sig. F Change
1	4	144	0.436

a. Predictors: (Constant), 性别，年龄，尿蛋白总量，滤过率

ANOVA[a]

Model		Sum of Squares	df	Mean Square	F	Sig.
1	Regression	1.038	4	0.260	0.952	0.436[b]
	Residual	39.266	144	0.273		
	Total	40.305	148			

a. Dependent Variable: 降幅

b. Predictors: (Constant), 性别，年龄，尿蛋白总量，滤过率

Coefficients[a]

Model		Unstandardized Coefficients B	Unstandardized Coefficients Std. Error	Standardized Coefficients Beta	t	Sig.
1	(Constant)	0.198	0.320		0.618	0.538
	尿蛋白总量	0.044	0.025	0.152	1.775	0.078
	滤过率	0.000	0.002	0.015	0.158	0.875
	年龄	0.003	0.004	0.072	0.793	0.429
	性别	-0.005	0.088	-0.005	-0.061	0.951

Coefficients[a]

Model		95.0% Confidence Interval for B Lower Bound	95.0% Confidence Interval for B Upper Bound	Collinearity Statistics Tolerance	Collinearity Statistics VIF
1	(Constant)	-0.434	0.829		
	尿蛋白总量	-0.005	0.094	0.920	1.087
	滤过率	-0.003	0.004	0.745	1.342
	年龄	-0.004	0.010	0.830	1.204
	性别	-0.180	0.169	0.940	1.064

a. Dependent Variable: 降幅

Collinearity Diagnostics[a]

Model	Dimension	Eigenvalue	Condition Index	Variance Proportions (Constant)	Variance Proportions 尿蛋白总量	Variance Proportions 滤过率
1	1	4.246	1.000	0.00	0.02	0.00
	2	0.508	2.890	0.00	0.84	0.01
	3	0.141	5.497	0.00	0.04	0.37
	4	0.091	6.815	0.00	0.00	0.00
	5	0.013	17.831	1.00	0.11	0.61

Collinearity Diagnostics[a]

Model	Dimension	Variance Proportions 年龄	Variance Proportions 性别
1	1	0.00	0.00
	2	0.00	0.00
	3	0.11	0.10
	4	0.32	0.58
	5	0.57	0.31

a. Dependent Variable: 降幅
